# Tannin-mediated improvement of* Moringa oleifera* silage: nutritional quality, aerobic stability, and methane mitigation

**DOI:** 10.1186/s12870-026-08507-9

**Published:** 2026-03-12

**Authors:** Zuhai Xu, Siming Ma, Yi Zhou, Xiaomin Wu, Jing Zhou, Fulin Yang

**Affiliations:** 1 College of Animal Sciences, Fujian Agriculture and Forestry University, Fuzhou, 350002 China; 2 Fujian Changle Sandy Habitat Chinese Herbal Medicine Science and Technology Backyard, Fuzhou, 350002 China; 3 College of Life Sciences, Fujian Agriculture and Forestry University, Fuzhou, 350002 China; 4 College of Resources and Environment, Fujian Agriculture and Forestry University, Fuzhou, 350002 China

**Keywords:** *Moringa oleifera*, Tannic acid, Gallic acid, Polyethylene glycol, Vitro methane emissions, Aerobic stability

## Abstract

**Background:**

Methane emissions during livestock production have become an important source of greenhouse gases. Silage feed, as the main feed source for ruminants, plays a significant role in the livestock industry.

**Methods:**

In this experiment, three different tannin-related compounds (tannic acid, gallic acid and polyethylene glycol) were added as silage additives to *Moringa oleifera* leaf silage. Their effects on the nutritional quality, fermentation quality, aerobic stability and microbial community of *Moringa oleifera* leaf silage were determined, and in vitro experiments were conducted to determine their influence on rumen methane emissions.

**Results:**

Showed that TA and GA increased dry matter and true protein content while reducing neutral detergent fiber, the ratio of non-protein nitrogen to total nitrogen, and ammonia nitrogen levels (*P* < 0.05). TA enhanced aerobic stability for the first three days of aerobic exposure, whereas the 1% GA treatment maintained a stable pH value throughout the aerobic exposure period (4.19–4.27). Both TA and GA significantly reduced in vitro methane emissions without compromising dry matter digestibility (*P* < 0.05), among them, the 1% GA group reduced methane emissions by 21.3% compared to the CK (32.67 ml vs 41.52 ml). In contrast, PEG promoted lactic acid bacteria growth and lactic acid accumulation while inhibiting undesirable microorganisms such as *Enterobacter*; however, its overall impact on silage preservation was limited.

**Conclusions:**

These findings suggest that 1% GA could serve as a sustainable silage additive, effectively improving silage quality and aerobic stability while reducing greenhouse gas emissions. This experiment indicates that tannin compounds, especially gallic acid, can be chosen as an additive for *Moringa oleifera* leaf silage, which is conducive to the sustainable development of the livestock industry.

**Supplementary Information:**

The online version contains supplementary material available at 10.1186/s12870-026-08507-9.

## Introduction

CH_4_ is the second most important greenhouse gas after CO₂ with a global warming potential approximately 28 times that of CO₂ and an atmospheric lifetime of about 12.2 years [1–3]. Livestock farming is one of the main sources of methane emissions, accounting for approximately 14.5% of global greenhouse gas emissions [4–7]. Approximately 87% of the intestinal methane emitted by ruminants comes from the rumen, and effective emission reduction strategies are urgently needed [8]. Meanwhile, the traditional grazing system has been unable to meet the growing feed demands, posing a threat to the sustainability of the global livestock industry [9]. Therefore, the industry is confronted with a dual challenge: while enhancing feed conversion efficiency, it must also take into account environmental sustainability [10]. To address this situation, there is an urgent need to explore and develop new alternative feed resources, especially natural woody plants rich in nutrients [11].

At present, the common woody plant feeds mainly include mulberry, paper mulberry, acacia, Ficus *Moringa oleifera*, etc. Among them, *Moringa oleifera* is the most promising multi-functional woody feed crop for development due to its more balanced nutritional quality, biological activity, stress resistance, processing adaptability and silage potential [12].

*Moringa oleifera* leaves are a nutrient-dense feed ingredient, with a protein content of up to 25.02% [13], and a wide array of vitamins and minerals. Their richness in phenolic and bioactive compounds confers antibacterial and pest-resistant properties, making *Moringa oleifera* leaves a viable alternative to conventional livestock feed [14–16]. In recent years, *Moringa oleifera* leaves has been widely adopted as a protein source to improve livestock health, growth performance, milk yield, and meat quality [17, 18]. Leitanthem et al. [19] demonstrated that replacing 10–20% of concentrate feed with *Moringa oleifera* leaves in goat diets enhanced digestibility, growth, immune function, and antioxidant capacity while reducing enteric methane emissions. Adegun and Aye [20] reported improved growth performance in sheep supplemented with *Moringa oleifera* leaves, and Zeng et al. [21] confirmed that *Moringa oleifera* leaves meal could fully replace corn silage in diets for lactating dairy cows without affecting milk yield or composition. However, fresh woody plant biomass typically has high moisture content. While haymaking increases lignification, it often leads to significant leaf loss during drying and substantial nutrient degradation [22]. In contrast, silage—a fermentation-based preservation method—converts fresh forage into stable, storable feed and is a key technology for utilizing woody plants [23]. A major challenge is that more than 50% of *Moringa oleifera*'s protein can degrade during ensiling [24], generating substantial non-protein nitrogen (NPN). Elevated NPN levels increase ruminal NH₃-N concentration, reducing nitrogen utilization efficiency and potentially increasing CH₄ emissions [25, 26]. Therefore, suitable silage additives are needed to improve the quality of silage.

Tannin application is a widely adopted strategy to mitigate protein degradation in the rumen or silage [27]. As polyphenolic compounds with aromatic-ring structures, tannins are categorized into hydrolyzable and condensed types based on their hydrolytic properties. Beyond binding proteins, tannins exhibit dual antibacterial mechanisms: (1) disrupting microbial cell membranes and causing lysis, and (2) complexing with cellular components (e.g., cell walls, membrane proteins, enzymes), thereby impairing microbial physiology and growth [28]. These actions can directly suppress methanogenic activity and indirectly reduce hydrogen substrate availability [29]. Castillo et al. [30, 31] demonstrated that tannic acid has the ability to improve the production performance of animals. By mixing condensed and hydrolyzable tannins and adding them to the diet of lactating cows, it can shorten the calving interval, save feed costs, and enhance the calving performance of the cows, increase milk production and milk quality.

Hydrolyzable tannins protect premium-grade proteins from ruminal and ensiling degradation through pH-reversible tannin-protein complexation, ensuring targeted nutrient release in the abomasum and small intestine [32]. Tannic acid (TA), a common hydrolyzable tannin, hydrolyzes into monomers under acidic or enzymatic conditions [33]. In vitro studies show that tannin hydrolysates possess unique antibacterial [34] and antioxidant properties [35]. Gallic acid (GA), a hydrolysis product of gallotannins, exhibits broad-spectrum antimicrobial activity. Aboagye et al. [36] found that moderate GA concentrations can reduce ruminal CH₄ emissions without compromising production performance. Both TA and GA bind proteins effectively, inhibiting degradation to improve silage quality [32]. Moreover, compared with TA, in addition to directly binding proteins, the degradation products of GA during silage, such as pyrogallic acid, resorcinol and resorcinol, also have the activity of inhibiting protein hydrolysis. However, the hydrolysis of tannic acid macromolecular structure is slow, and the synergistic effect of degradation products is relatively limited. At the same time, in vitro rumen fermentation experiments have shown that The yield of volatile fatty acids (VFA) in the GA treatment was higher than that in the TA treatment, and the NH_3_-N concentration also increased, indicating that the gallic acid-protein complex is more easily dissociated and utilized by microorganisms in the rumen [37]. The latest research has achieved the batch production of gallic acid using glucose as the raw material in the Escherichia coli culture system, which has significantly reduced the production cost of gallic acid and brought higher economic benefits to the practical application of gallic acid as a silage additive in production [38]. Polyethylene glycol (PEG) can form a stable hydrogen-bonded complex (PEG-CT) with endogenous condensed tannins (CT), making it a widely employed tannin inhibitor and a valuable tool for investigating the role of CT in silage fermentation [39]. Previous research confirms that 1% and 2% TA and GA can enhance protein preservation and antioxidant activity in *Moringa oleifera* leaves silage [40]. Based on this, in this experiment, 0.5% TA and GA were additionally set to further investigate the effects of low concentrations of TA and GA on the quality of *Moringa oleifera* leaves silage. Meanwhile, PEG as a condensed tannin inactivator in other silages, their impacts on the aerobic stability of *Moringa oleifera* leaves silage and associated ruminal CH₄ production remain unexplored.

So, this study treated *Moringa oleifera* leaves silage with TA, GA, and PEG to evaluate their effects on silage quality, in vitro CH₄ emissions, and aerobic stability, thereby addressing key gaps in sustainable feed innovation. We hypothesized that TA and GA would reduce protein degradation through tannin-protein complexation, while PEG would negate intrinsic tannin effects. Our findings offer a potential strategy for optimizing *Moringa oleifera* leaves utilization, reducing enteric methane emissions, and advancing sustainable livestock production. Future research should investigate ruminal microbial community dynamics during *Moringa oleifera* leaves digestion to further elucidate the mechanisms underlying methane production.

## Methods

### Plant materials and silage additives

*Moringa oleifera* leaves was harvested in July 2023 from the Fujian Changle Sandy Habitat Chinese Herbal Medicine Science and Technology Backyard, operated by Fujian Binhai Biotechnology Co., Ltd., in Xiuyuan Baikao Recreation Town, (N 25.96°, E 119.5°; Fuzhou, China). Species identification followed standard taxonomic descriptions, and no field collection of wild plant materials was involved. A voucher specimen (FAFU-CAS-202401) has been deposited in the College of Animal Sciences, Fujian Agriculture and Forestry University All experimental procedures complied with institutional and national guidelines. The harvested material had a moisture content of 74.4%. The *Moringa oleifera* leaves was selected, cut into 2–3 cm pieces, mixed, and then wilted naturally for approximately 8 h until the moisture content reaches the range suitable for ensiling. The additives, TA, GA, and polyethylene glycol 6000 (PEG6000), were purchased from McLean Reagent (Shanghai, China). The prepared *Moringa oleifera* leaves was thoroughly mixed, and the additives were applied as follows: the control (CK) received an equal volume of sterile water, while the treatment groups received TA at 0.5%, 1%, or 2% (TA1, TA2, TA3), GA at 0.5%, 1%, or 2% (GA1, GA2, GA3) or PEG at 5%, 10%, or 20% (PEG1, PEG2, PEG3) (all w/w based on fresh weight). The selection of PEG concentration is based on the research conducted by Ali Hatami et al. [41]. Additives were sprayed onto the *Moringa oleifera* leaves using a micro-sprayer and mixed thoroughly to ensure uniformity Subsequently, 400 g of the treated material was packed into each vacuum-sealed bag (24 cm × 35 cm). A total of sixty bags were randomly assigned to the ten treatment groups, with each group having two ensiling durations (30 and 60 days) and three replicates. All bags were stored at 25 ± 2 °C for the ensiling period. This temperature is close to the annual average temperature in southern China.

### Silage quality analysis

The dry matter (DM) content of *Moringa oleifera* leaves was determined by drying in an oven at 65 ℃ for 72 h; Water-soluble carbohydrates (WSC) were determined using the anthrone–sulfuric acid colorimetric method [42]; Acid detergent fiber (ADF) and Neutral detergent fiber (NDF) contents were determined according to Van Soest’s method [43], using a fully automated fiber analyzer (FIWE Advance, Bepure Scientific Instruments Co., Ltd., Beijing, China); Hemicellulose (HC) content was calculated as the difference between NDF and ADF. Total nitrogen (TN) content was determined using an automatic Kjeldahl apparatus (Haineng, Jinan China) and Crude protein (CP) content was calculated as TN × 6.25. True protein (TP) was determined by the trichloroacetic acid precipitation method, and NPN was calculated as the difference between CP and TP. *Moringa oleifera* leaves silage samples (20 g each) were homogenized with 180 mL of distilled water in a blender for 1 min, filtered through four layers of gauze, and the resulting filtrates were used for analyses of pH, ammonia nitrogen (NH_3_-N), and organic acids. pH was determined using a pHS-3D acidimeter (Leici, Shanghai, China); Lactic acid (LA) content was determined using the p-hydroxybenzene colorimetric method [44]; NH_3_-N content was calculated by phenol-sodium hypochlorite colorimetric method. The bacterial populations of lactic acid bacteria (LAB), yeasts, molds and aerobic bacteria (AB) were counted after being cultured for 2 days at 30℃ on MRS agar, malt extract agar and plate count agar. Concentrations of acetic acid (AA), propionic acid (PA), and butyric acid (BA) were analyzed by gas chromatography using an Agilent 7890 A system. (Agilent7890A, Beijing, China).

### The aerobic stability analysis

After 60 days of fermentation, *Moringa oleifera* leaves silage samples were unsealed and exposed to environmental temperature of 25℃ for 9 days to evaluate aerobic stability. On days 0, 3, 6, and 9 of aerobic exposure, three replicate samples were collected from each silage treatment group and analyzed for pH, LA, NH₃–N, and AA content.

### Microbial community determination

Based on the fermentation quality assessment, the optimal additive concentrations were identified as TA3, GA2, and PEG3. Subsequently, fresh matter (FM) and the corresponding silage samples from these selected treatments were homogenized, and three replicate subsamples were prepared from each. Approximately 10 g of each subsample was placed into a 50 mL sterile centrifuge tube, snap-frozen in liquid nitrogen for 10 min, and then transferred to −80 °C for long-term storage.

This assay encompassed five treatment groups, with three replicate samples randomly selected from each group for high-throughput sequencing analysis. DNA extraction was performed using the cetyltrimethylammonium bromide (CTAB) protocol. The purity, concentration, and integrity of DNA were assessed with a NanoDrop2000 instrument via 1% agarose gelelectrophoresis. After the DNA samples underwent amplification, the PCR products were subsequently mixed, purified, and processed through a series of meticulous steps including end repair, A-tailing, and the addition of sequencing adapters. The DNA samples were sequenced using the primer sequences 315 F (5′-CCTAYGGGRBGSCAG-3′) and 806R (5′-GGACTACNNGGGTATCTAAT-3′) was used to PCR amplify the 16S rDNA gene of the highly variable region of bacteria V3 ~ V4. The PCR amplified products were identified by agarose gel electrophoresis, then subjected to magnetic bead purification and aliquoted according to the concentration, and then assayed and recovered for the target bands after mixing. Miseq library was constructed using NEXTFLEXRapidDNA-SeqKit and sequenced. Upon completion of library construction, the quantity of the library was assessed using both Qubit and Q-PCR methodologies. The PE250 was up-sequenced using NovaSeq6000 (Illumina, America) and the sequencing was completed by Beijing Novo (Beijing, China). After Reads splicing, Tags filtering, de-chimerization and amplicon sequence variants (ASVs). clustering noise reduction analysis, species annotation and characteristic sequence analysis were performed. The α-diversity and β-diversity analyses elucidated the variations in the microbial species composition within each group and the community structure between groups before and after *Moringa oleifera* leaves silage. The raw sequencing data from this experiment have been uploaded to the National Center for Biotechnology Information’s database with accession numbers PRJNA1372840.

### Determination of in vitro digestibility and methane emissions

Rumen fluid was collected from three adult male Dehua black mountain goats (average body weight: 33 kg) immediately after slaughter from slaughter-house. All the goats were fed the same kind of feed. The formulation of the total mixed ration includes 30% peanut vine, 20% elephant grass, 15% sweet potato vine, 20% corn, 5% bran, 5% soybean meal, 2% cottonseed meal and 3% minerals and additives. A rumen buffer was prepared according to the method of Menke et al. [45]. Prior to morning feeding, rumen contents were collected from various locations within the rumen, pooled, and filtered through four layers of sterile gauze. The filtered rumen fluid was then mixed with pre-warmed (39 °C) buffer at a ratio of 1:2 (v/v) to prepare the fermentation inoculum. For the in vitro fermentation, 2.5 g of each sample was accurately weighed into a nylon bag. Each bag was placed into a 500 mL anaerobic fermentation vessel of a MultiTalent PX3 automated rumen simulation fermentation system (Bepure Scientific Instruments Co., Ltd., Beijing, China). Then, 400 mL of the freshly prepared rumen fermentation inoculum was added to each vessel. The headspace of each vessel was flushed with CO₂ for 30 s to establish anaerobic conditions, followed by incubation at 39 °C for 48 h. Gas production was automatically monitored and recorded throughout the incubation. After fermentation, the IVDMD was determined. The methane production data were fitted using a modified Gompertz growth equation model [46] and the equation was optimized:

1 $$\mathrm{V(t)} = \mathrm{V}_{(\infty)} \times \exp(-\exp(-\mathrm{K} \times (\mathrm{t} - \mathrm{X}_{c})))$$

where V(t) is the cumulative gas emission (ml); V_(∞)_ is the maximum cumulative gas emission (ml); K is the maximum gas emission rate (ml/h); X_c_ is the lag time (h); t is the time (h).

Relative feeding value (RFV), Total digestible nutrient (TDN), Relative feed quality (RFQ) are calculated using the following formulas respectively.

2 $$\mathrm{RFV} = (\mathrm{DDM} \times \mathrm{DMI})\, /\,1.29$$

3 $$\mathrm{TDN,}\, \% = 82.38 - (0.7515 \times \%\mathrm{ADF})$$

4 $$\mathrm{RFQ} = (\mathrm{DMI} \times \mathrm{TDN})\,/\, 1.23$$

where DDM represents the content of digestible DM. DDM, % = 88.9 – (0.779 × %ADF), DMI represents the expected dry matter intake. DMI (BW%) = 120/%NDF.

### Statistical analysis

The basic data analysis and organization were completed using Excel 2019 software. All data were statistically analyzed using SPSS 26.0 software. Before conducting general linear model analysis, normality tests and homogeneity of variance tests are first performed on the data. The normality test was conducted using the Shapiro–Wilk test, and a comprehensive judgment was made in combination with the Q-Q graph. The homogeneity of variance test was conducted using the Levene test to evaluate whether the variances among the treatment groups were equal. If the data satisfy normality or homogeneity of variance, a general linear model is used to analyze and test the effects of additives, silage days and their interactions on each measured index, and the Tukey HSD method is used for multiple comparisons. If the data does not satisfy normality or homogeneity of variance, appropriate transformation of the data should be carried out before analysis. If the conversion still does not meet the requirements, the Scheirer-Ray-Hare test is used instead of the parameter test. The results are expressed as mean ± standard deviation. *P* < 0.05 indicates a significant difference, and *P* < 0.01 indicates a highly significant difference. Correlation plots were generated using R (version 4.4.3) and Origin 2024.

## Results and discussion

### Raw material characteristics of Moringa oleifera leaves

The growth and reproduction of LAB are influenced by the moisture content, WSC availability, and buffering capacity of the forage. In this study, the DM content of *Moringa oleifera* leaves used for silage was 256.94 g/kg (Table S1), which is below the recommended range of 300–350 g/kg DM for ideal ensiling [47]. The CP content of *Moringa oleifera* leaves (158.92 g/kg DM) aligned with the 10–30% DM range reported by Kashyap et al. [48]. However, it was significantly lower than the 29.40% DM noted by Padayachee and Baijnath [49]. These observed discrepancies may be attributed to variations in the plant material's growth stage and differences in growing conditions. WSC content and feedstock microbial epiphytic community are pivotal factors influencing silage fermentation. It is well established that effective silage fermentation requires a WSC content of at least 50 g/kg DM [50]. In this study, the WSC content was 73.99 g/kg DM, which satisfies this requirement. Nevertheless, the epiphytic LAB population in the fresh material was 3.26 log CFU/g, may be insufficient to reach the optimal threshold (≥ 5.0 log CFU/g) for effective silage fermentation [51]. Therefore, the natural silage of *Moringa oleifera* leaves likely exhibited suboptimal fermentation quality, attributable to its high moisture content, low initial LAB count, and the presence of competing aerobic bacteria (3.54 log₁₀ CFU/g) and yeasts (3.82 log₁₀ CFU/g).

### Additives improve nutritional quality

This study investigated the impact of different additives on the nutritional quality of *Moringa oleifera* leaves silage. The experimental results show that compared with the DM content of fresh *Moringa oleifera* leaves (256.94 g/kg), each group had varying degrees of DM content loss after ensiling. However, compared with the CK, each group significantly reduced this loss (*P* < 0.05, Tables 1– 3). Moreover, DM content increased progressively with rising additive concentration, indicating a dose-dependent improvement in preservation efficiency. Notably, ensiling duration (30 vs. 60 days) did not significantly affect DM content within the same treatment. This stability is likely due to the combined action of *Moringa oleifera* leaves endogenous tannins and additive-induced suppression of detrimental microbial activity, which collectively minimized dry matter loss. With the progression of the ensiling period, the WSC content exhibited a significant decline (*P* < 0.05, Tables 1– 3) in all groups except for TA1, primarily due to cellular respiration, microbial activity, and the decomposition of fibers. After 30 days, WSC levels in TA1 (21.69 g/kg DM) and PEG3 (20.32 g/kg DM) were not significantly different from the CK (15.98 g/kg DM; *P* > 0.05), whereas other treatments significantly elevated WSC content (*P* < 0.05). This elevation may be linked to enhanced degradation of HC and NDF in the TA and GA groups, which released WSC. The higher fiber degradation in the TA1 group still resulted in lower WSC content, probably because lower concentrations of TA were not sufficient to inhibit the fermentation of microorganisms in silage that use WSC as a substrate. Similarly, the high PEG dosage may have diluted fermentable substrates, limiting WSC accumulation.

**Table 1:** Effect of TA on quality of MOL silage

Items	CK		TA1		TA2		TA3		*P-*value		
	30 d	60 d	30 d	60 d	30 d	60 d	30 d	60 d	D	T	D × T
pH	4.49 ± 0.04^Ab^	4.81 ± 0.07^B^	4.64 ± 0.04^Ba^	4.82 ± 0.03^A^	4.44 ± 0.04^Bb^	4.76 ± 0.08^A^	4.44 ± 0.01^Bb^	4.70 ± 0.09^A^	0.000	0.053	0.589
LA (g/kg DM)	12.78 ± 0.06^Aa^	5.62 ± 0.24^Bb^	9.83 ± 0.23^Ab^	6.60 ± 0.42^Bb^	12.37 ± 0.34^Aa^	5.75 ± 0.08^Bb^	7.77 ± 0.09^c^	8.98 ± 1.57^a^	0.000	0.058	0.000
AA (g/kg DM)	3.82 ± 0.26^b^	5.16 ± 0.18^b^	4.69 ± 0.24^a^	6.17 ± 0.33^a^	4.22 ± 0.12^ab^	5.67 ± 0.29^ab^	4.06 ± 0.17^ab^	5.35 ± 0.32^ab^	0.000	0.006	0.975
PA (g/kg DM)	0.12 ± 0.00	0.15 ± 0.01^a^	0.12 ± 0.00	0.14 ± 0.00^ab^	0.11 ± 0.00	0.13 ± 0.00^ab^	0.07 ± 0.04	0.13 ± 0.00b	0.006	0.112	0.645
BA (g/kg DM)	ND	0.16 ± 0.01	ND	0.14 ± 0.07	ND	0.12 ± 0.06	ND	0.12 ± 0.07	-	-	-
NH_3_-N (g/kg DM)	1.71 ± 0.07^a^	1.48 ± 0.09^a^	0.54 ± 0.04^Bd^	1.54 ± 0.06^Aa^	0.89 ± 0.06^Bc^	1.41 ± 0.02^Aa^	1.40 ± 0.01^Ab^	0.93 ± 0.02^Bb^	0.000	0.000	0.000
LAB (log CFU/g FM)	6.42 ± 0.02^Aa^	5.20 ± 0.04^Bb^	6.72 ± 0.13^Aa^	5.40 ± 0.11^Bb^	6.11 ± 0.08^Ab^	5.73 ± 0.11^Ba^	6.07 ± 0.05^Ab^	5.78 ± 0.06^Ba^	0.000	0.042	0.000
Yeast (log CFU/g FM)	< 2.00	3.46 ± 0.06^a^	< 2.00	2.51 ± 0.02^b^	< 2.00	2.45 ± 0.13^b^	< 2.00	2.11 ± 0.02^c^	-	-	-
AB (log CFU/g FM)	< 2.00	3.15 ± 0.02^a^	< 2.00	2.25 ± 0.03^b^	< 2.00	2.42 ± 0.19^b^	< 2.00	2.38 ± 0.11^b^	-	-	-
Mold (log CFU/g FM)	< 2.00	< 2.00	< 2.00	< 2.00	< 2.00	< 2.00	< 2.00	< 2.00	-	-	-
DM (g/kg FM)	236.75 ± 3.00^d^	236.25 ± 0.63^c^	248.42 ± 0.22^c^	248.38 ± 2.30^b^	254.83 ± 0.70^b^	252.33 ± 0.96^a^	263.50 ± 0.95^a^	248.92 ± 2.54^b^	0.101	0.000	0.450
WSC (g/kg DM)	20.32 ± 0.61^Ac^	15.12 ± 0.54^Bb^	21.70 ± 1.07^c^	18.94 ± 0.26^a^	24.47 ± 0.35^Ab^	15.21 ± 0.26^Bb^	35.17 ± 0.36^Aa^	13.74 ± 0.68^Bb^	0.000	0.000	0.000
NDF (g/kg DM)	555.28 ± 2.31^Aa^	518.40 ± 7.09^B^	547.83 ± 1.64^Aa^	506.95 ± 3.27^B^	550.19 ± 5.94^Aa^	510.81 ± 5.97^B^	521.44 ± 3.20^Ab^	509.70 ± 2.76^B^	0.000	0.000	0.079
ADF (g/kg DM)	382.80 ± 5.62^b^	363.05 ± 8.58^b^	426.76 ± 4.10^Aa^	377.00 ± 3.50^Bb^	417.89 ± 3.03^Aa^	383.02 ± 3.69^Bab^	417.57 ± 8.12^a^	399.35 ± 3.70^a^	0.000	0.000	0.030
HC (g/kg DM)	172.47 ± 7.77^a^	155.35 ± 15.12^a^	121.07 ± 3.60^b^	129.95 ± 6.76^ab^	132.29 ± 8.93^b^	127.79 ± 8.01^ab^	103.87 ± 18.45^b^	103.30 ± 6.89^b^	0.604	0.000	0.546

T Treatment, D Ensilage days, T × D interaction between treatment and ensilage days

Different capital letters in the same industry indicate significant differences in different group at the same time (*P* < 0.05), and different lowercase letters in the same column indicate significant differences in the same group at different times (*P* < 0.05)

The primary factor constraining the efficiency of feed utilization in ruminants is fiber digestibility, a high fiber content in feed may impede the absorption of other essential nutrients by ruminants [52]. With prolonged ensiling, NDF content decreased significantly (*P* < 0.05) in the TA treatments (Table 1), as well as in the PEG1 and PEG3 treatments (Table 3). Conversely, NDF content in both GA and PEG2 treatments remained stable during later silage stages. The different results presented by TA and GA in terms of time effect may be due to the difference in their molecular weights. During the silage process, TA may be hydrolyzed by tannase and esterase, releasing small molecule substances and carbohydrates, which indirectly promotes the growth of fiber-degrading bacteria. Moreover, the low pH environment caused by silage with GA as an additive further inhibits the fiber-degrading microorganisms, thereby slowing down the degradation of NDF over time. Elevated levels of NDF (> 55%) can compromise feed palatability and reduce animal intake. Thus, a decline in NDF serves as a dependable indicator of superior silage quality [53]. The fiber content remained at a relatively high level after ensiling, suggesting that ensiling alone has limited capacity for fiber degradation, and other methods need to be combined to improve efficiency [54]. At 30 days of ensiling, the NDF levels in TA3 and GA treated silages dropped significantly relative to the CK (*P* < 0.05, Tables 1 and 2). The decline presumably resulted from TA, GA, and fermentation-derived organic acids compromising lignocellulose integrity—organic acids break down the glycosidic bonds and ether bonds in the lignocellulosic matrix, thereby exposing more cellulose [55]. After 60 days of ensiling, the NDF content in the GA1 treatments was significantly lower (*P* < 0.05) compared with CK (Table 2). HC content was significantly lower (*P* < 0.05) in TA3 and GA1 treatments (Tables 1 and 2) across varying fermentation periods. On day 30 of ensiling, the ADF content showed a significant increase (*P* < 0.05) in all TA groups and the PEG2 group compared to CK. By day 60, ADF levels remained significantly elevated (*P* < 0.05) in the TA3, GA1, and PEG2 treatments relative to CK (Tables 1– 3). This might be because the cellulose and lignin constituting ADF have stable chemical properties and are difficult to be degraded by other compounds [56].

**Table 2:** Effect of GA on quality of MOL silage

Items	CK		GA1		GA2		GA3		*P-*value		
	30 d	60 d	30 d	60 d	30 d	60 d	30 d	60 d	D	T	D × T
pH	4.49 ± 0.04^Ab^	4.81 ± 0.07^B^	4.64 ± 0.04^Ba^	4.82 ± 0.03^A^	4.44 ± 0.04^Bb^	4.76 ± 0.08^A^	4.44 ± 0.01^Bb^	4.70 ± 0.09^A^	0.000	0.053	0.589
LA (g/kg DM)	12.78 ± 0.06^Aa^	5.62 ± 0.24^Bb^	9.83 ± 0.23^Ab^	6.60 ± 0.42^Bb^	12.37 ± 0.34^Aa^	5.75 ± 0.08^Bb^	7.77 ± 0.09^c^	8.98 ± 1.57^a^	0.000	0.058	0.000
AA (g/kg DM)	3.82 ± 0.26	5.16 ± 0.18^a^	3.51 ± 0.36	3.84 ± 0.10^b^	3.52 ± 0.16	3.48 ± 0.05^b^	3.74 ± 0.05	4.37 ± 0.48^ab^	0.006	0.006	0.078
PA (g/kg DM)	0.12 ± 0.00	0.15 ± 0.01^a^	0.11 ± 0.00	0.12 ± 0.00^b^	0.11 ± 0.00	0.11 ± 0.00^b^	0.08 ± 0.04	0.13 ± 0.00^b^	0.032	0.169	0.321
BA (g/kg DM)	ND	0.16 ± 0.01	ND	0.14 ± 0.07	ND	ND	ND	0.07 ± 0.07	-	-	-
NH_3_-N (g/kg DM)	1.71 ± 0.07^a^	1.48 ± 0.09^a^	1.23 ± 0.07^b^	0.97 ± 0.08^b^	0.88 ± 0.02^c^	0.80 ± 0.09^b^	0.47 ± 0.07^d^	0.76 ± 0.04^b^	0.162	0.000	0.000
LAB (log CFU/g FM)	6.42 ± 0.02^Aa^	5.20 ± 0.04^Bb^	6.11 ± 0.04^b^	5.64 ± 0.23^b^	5.79 ± 0.06^Bc^	6.24 ± 0.09^Aa^	5.22 ± 0.07^d^	5.36 ± 0.08^b^	0.010	0.000	0.000
Yeast (log CFU/g FM)	< 2.00	3.46 ± 0.06^a^	< 2.00	< 2.00	< 2.00	< 2.00	< 2.00	< 2.00	-	-	-
AB (log CFU/g FM)	< 2.00	3.15 ± 0.02^a^	< 2.00	< 2.00	< 2.00	< 2.00	< 2.00	< 2.00	-	-	-
Mold (log CFU/g FM)	< 2.00	< 2.00	< 2.00	< 2.00	< 2.00	< 2.00	< 2.00	< 2.00	-	-	-
DM (g/kg FM)	236.75 ± 3.00^c^	236.25 ± 0.63^c^	250.33 ± 3.31^b^	250.27 ± 0.24^ab^	250.50 ± 2.78^b^	249.30 ± 2.98^b^	267.68 ± 2.95^a^	258.58 ± 2.63^a^	0.180	0.000	0.340
WSC (g/kg DM)	20.32 ± 0.61^Ab^	15.12 ± 0.54^Ba^	30.84 ± 2.01^Aa^	17.03 ± 0.57^Ba^	26.31 ± 0.34^Aa^	14.07 ± 0.91^Ba^	28.24 ± 0.33^Aa^	9.70 ± 0.70^Bb^	0.000	0.000	0.000
NDF (g/kg DM)	555.28 ± 2.31^Aa^	518.40 ± 7.09^Ba^	478.37 ± 5.43^c^	480.35 ± 6.40^b^	513.89 ± 8.09^b^	499.59 ± 5.02^ab^	522.66 ± 6.97^b^	509.02 ± 4.94^a^	0.002	0.000	0.039
ADF (g/kg DM)	382.80 ± 5.62	363.05 ± 8.58^bc^	383.27 ± 4.08	387.39 ± 4.50^a^	376.31 ± 4.01^A^	357.24 ± 2.46^Bc^	378.44 ± 6.30	381.40 ± 2.58^ab^	0.044	0.013	0.046
HC (g/kg DM)	172.47 ± 7.77^a^	155.35 ± 15.12^a^	95.09 ± 5.74^b^	92.96 ± 10.90^b^	137.58 ± 11.73^a^	142.35 ± 7.46^a^	144.22 ± 12.13^a^	127.62 ± 2.53^ab^	0.280	0.000	0.620

*T* Treatment, *D* Ensilage days; T × D interaction between treatment and ensilage days

Different capital letters in the same industry indicate significant differences in different group at the same time (*P* < 0.05), and different lowercase letters in the same column indicate significant differences in the same group at different times (*P* < 0.05)

**Fig. 1:**
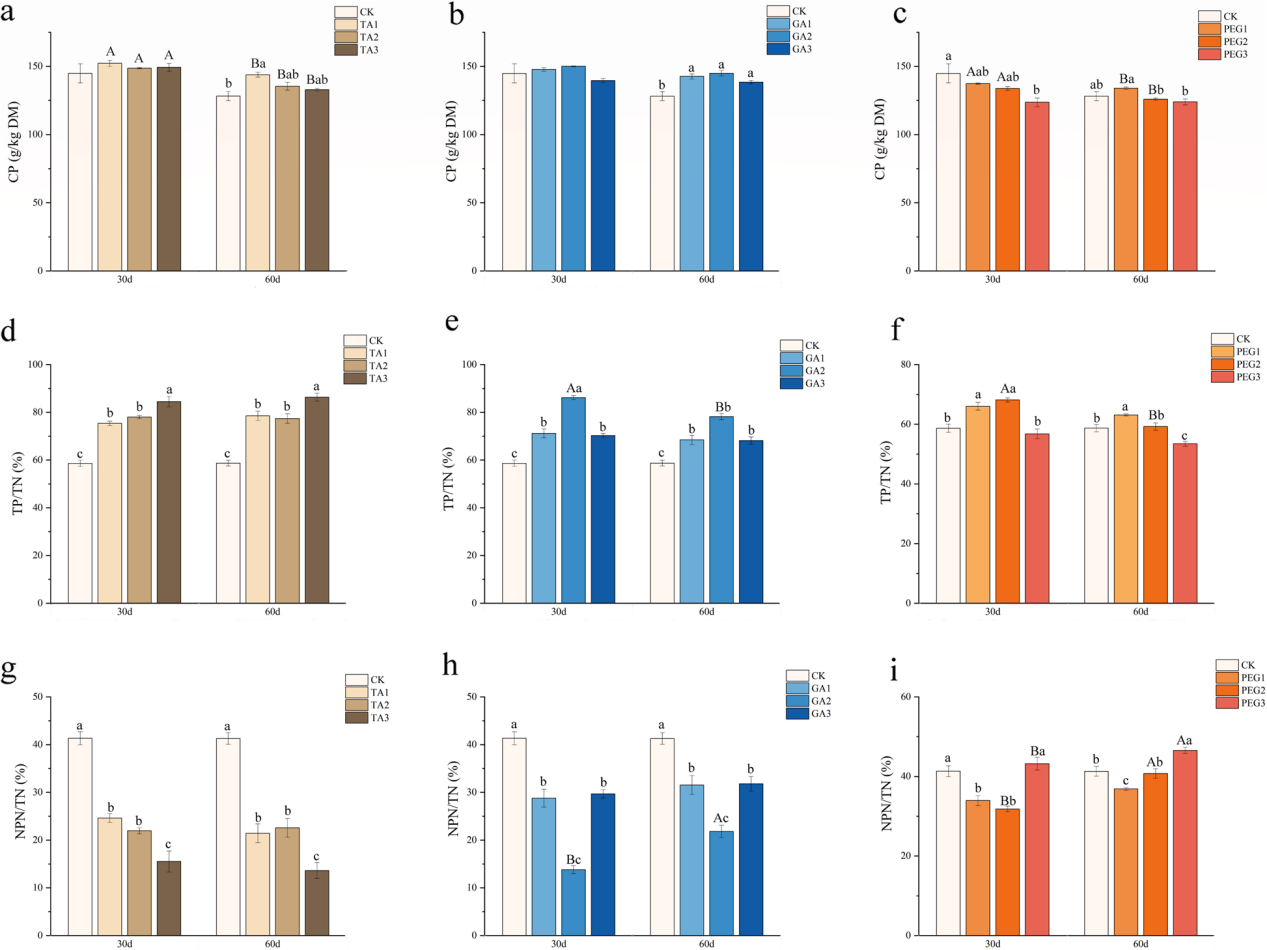
Effects of three additives on Protein composition. **a, b, c** Effect of three additives on CP. **d, e, f** Effect of three additives on TP/TN. **g, h, i** Effect of three additives on NPN/TN. Different capital letters in the same industry indicate significant differences in different group at the same time (*P* < 0.05), and different lowercase letters in the same column indicate significant differences in the same group at different times (*P* < 0.05). The same below

The ensiling process involves two distinct phases of protein hydrolysis that yield free amino acids. The initial phase is characterized by plant protease-mediated catabolism, where proteins are cleaved into free amino acids and peptides. This is followed by microbial intervention, which accelerates further protein breakdown through deamination, yielding various byproducts such as amides, amines, and ammonia [57]. The experimental results showed that the CP content in the TA, PEG1 and PEG2 treatments decreased significantly (*P* < 0.05, Fig. 1a–c) with increasing ensiling time. At 60 days, both TA1 and GA groups maintained significantly higher CP content relative to CK (*P* < 0.05, Fig. 1a–b). The superior CP preservation in the GA treatment may be attributed to multiple factors: (1) the antimicrobial and protease-inhibitory properties of GA, and (2) the lower pH achieved during ensiling, which collectively suppressed excessive proteolysis and deamination. As a result, less protein was converted to ammonia, leading to higher residual CP [58]. In previous reports, compared with other silage additives that play a role in protein preservation (Bacillus subtilis, cellulase), GA also demonstrated better silage effects, manifested as higher LA and WSC contents as well as a more reasonable bacterial community structure [59].

For ruminants, NPN is utilized less efficiently than TP. As a result, excessive protein hydrolysis during fermentation leads to reduced silage nutritional value [60]. In this study, the addition of different concentrations of TA and GA increased the TP/TN and decreased the NPN/TN ratios in *Moringa oleifera* leaves silage (Fig. 1d, e, g, h), confirming that TA and GA supplementation effectively mitigates TP degradation in *Moringa oleifera* leaves silage. Among them, the TP/TN ratio in the TA3 and GA2 treatments was significantly elevated (*P* < 0.05) compared to the other treatments within each respective group. By enhancing protein preservation quality, these additives reduce nitrogen loss while minimizing environmental impact. Consistent with prior research, after 30 days of ensiling, TA demonstrates superior protein preservation capability compared to GA. This advantage likely stems from TA's distinct molecular structure and higher polyphenol content, which facilitate the formation of more stable conjugates with a greater number of protein molecules [61]. In contrast the addition of PEG significantly improved the generation of NPN in silage. This enhancement is likely attributed to its role in promoting undesirable bacteria fermentation [62].

### TA and GA slow down fermentation during silage

We further assessed the fermentation quality of *Moringa oleifera* leaves silage. After 30 days of ensiling, none of the treatment groups achieved the ideal pH, likely due to the inherent recalcitrance of the raw material to fermentation and the antimicrobial effects of the additives. Among all treatments, the GA group (particularly GA2) exhibited pH values closest to the optimal range for silage. Notably, during prolonged fermentation, the pH increased significantly (*P* < 0.05) in the CK, TA, and PEG groups (Tables 1 and 3). TA-treated silage consistently displayed higher pH than the GA group. This distinction may stem from: (1) inherent differences in acidity and antimicrobial potency between the additives, (2) differential inhibition of protein degradation by TA and GA during ensiling, which could subsequently alter the buffering capacity of the silage matrix.

**Table 3:** Effect of PEG on quality of MOL silage

Items	CK		PEG1		PEG2		PEG3		*P-*value		
	30 d	60 d	30 d	60 d	30 d	60 d	30 d	60 d	D	T	D × T
pH	4.49 ± 0.04^Ba^	4.81 ± 0.07^A^	4.49 ± 0.03^Ba^	4.94 ± 0.05^A^	4.49 ± 0.04^Ba^	4.86 ± 0.03^A^	4.24 ± 0.02^Bb^	4.90 ± 0.01^A^	0.000	0.267	0.639
LA (g/kg DM)	12.78 ± 0.06^A^	5.62 ± 0.24^Bb^	12.78 ± 0.06	11.24 ± 0.24^a^	12.94 ± 0.05	11.39 ± 0.10^a^	12.24 ± 0.48	11.76 ± 0.13^a^	0.000	0.000	0.000
AA (g/kg DM)	3.82 ± 0.26	5.16 ± 0.18	4.24 ± 0.34	5.73 ± 0.22	5.16 ± 0.69	5.64 ± 0.14	4.52 ± 0.33	5.95 ± 0.29	0.000	0.085	0.447
PA (g/kg DM)	0.12 ± 0.00	0.15 ± 0.01	0.12 ± 0.00	0.15 ± 0.01	0.15 ± 0.01	0.15 ± 0.00	0.12 ± 0.00	0.15 ± 0.00	0.567	0.416	0.405
BA (g/kg DM)	ND	ND	ND	ND	0.04 ± 0.04	ND	ND	ND	-	-	-
NH_3_-N (g/kg DM)	1.71 ± 0.07^a^	1.48 ± 0.09^a^	1.09 ± 0.02^c^	1.75 ± 0.08	1.32 ± 0.01^b^	1.67 ± 002	1.41 ± 0.05^b^	1.75 ± 0.11	0.000	0.000	0.000
LAB (log CFU/g FM)	6.42 ± 0.02^Ac^	5.20 ± 0.04^Bc^	7.56 ± 0.02^Aa^	6.46 ± 0.09^Bb^	7.35 ± 0.08^Bb^	7.69 ± 0.07^Aa^	7.31 ± 0.02^b^	7.36 ± 0.17^a^	0.000	0.000	0.000
Yeast (log CFU/g FM)	< 2.00	3.46 ± 0.06^a^	< 2.00	3.25 ± 0.03^b^	< 2.00	3.25 ± 0.02^b^	2.51 ± 0.13	3.35 ± 0.01^ab^	-	-	-
AB (log CFU/g FM)	< 2.00	3.15 ± 0.02^b^	< 2.00	3.07 ± 0.05^b^	< 2.00	3.34 ± 0.05^a^	< 2.00	3.12 ± 0.06^b^	-	-	-
Mold (log CFU/g FM)	ND	< 2.00	< 2.00	< 2.00	< 2.00	< 2.00	< 2.00	< 2.00	-	-	-
DM (g/kg FM)	236.75 ± 3.00^c^	236.25 ± 0.63^c^	251.25 ± 1.28^b^	245.00 ± 0.76^b^	255.92 ± 2.69^b^	250.83 ± 3.52^b^	266.83 ± 1.25^a^	259.27 ± 2.66^a^	0.005	0.000	0.380
WSC (g/kg DM)	20.32 ± 0.61^Ab^	15.12 ± 0.54^Ba^	31.34 ± 1.53^Aa^	10.27 ± 0.4^Bc^	35.26 ± 1.87^Aa^	11.20 ± 0.48^Bbc^	15.98 ± 0.11^Ab^	12.82 ± 0.40^Bb^	0.000	0.000	0.000
NDF (g/kg DM)	555.28 ± 2.31^A^	518.40 ± 7.09^B^	534.13 ± 6.58^A^	478.84 ± 9.22^B^	551.05 ± 13.70	514.25 ± 5.32	537.52 ± 13.76^A^	485.63 ± 9.46^B^	0.000	0.030	0.750
ADF (g/kg DM)	382.80 ± 5.62^b^	363.05 ± 8.58^b^	396.94 ± 8.02^b^	368.57 ± 12.14^ab^	427.46 ± 3.91^Aa^	397.39 ± 2.21^Ba^	387.19 ± 2.83^Ab^	350.99 ± 5.23^Bb^	0.000	0.000	0.690
HC (g/kg DM)	172.47 ± 7.77^a^	155.35 ± 15.12^a^	137.19 ± 13.18	110.27 ± 17.31c	123.59 ± 16.04	116.86 ± 19.71	150.33 ± 14.67^b^	134.63 ± 10.23	0.130	0.033	0.920

*T* Treatment, *D* Ensilage days, T × D interaction between treatment and ensilage days

Different capital letters in the same column represent significant differences between different silage times (*P* < 0.05), and different lowercase letters in the same column represent significant differences between different concentration treatment groups (*P* < 0.05)

As the primary byproduct of LAB fermentation, LA rapidly lowers pH, creating an optimal acidic environment for silage preservation. This not only reduces nutrient degradation but also enhances aerobic stability [63]. An acidic environment characterized by a low pH exerts a detrimental impact on the cell membrane and enzymatic systems of deleterious microorganisms, thus inhibiting their growth and reproduction [64]. Previous studies have demonstrated that hydrolyzable tannin supplementation in feed reduces LA content while elevating pH [65]—a pattern consistent with our observations during the 30-day TA treatment phase. After 30 days of ensiling, the LA content in the TA group was reduced compared to the CK, whereas the GA1 and GA3 treatments significantly increased the LA content (*P* < 0.05, Tables 1 and 2). Notably, a portion of the LA initially produced was further metabolized by microorganisms between days 30 and 60 of ensiling, being converted into acetic acid, propionic acid, and other metabolites. By day 60, no significant LA content differences were observed among the TA1, TA2, and CK groups (*P* > 0.05). PEG treatments significantly elevated LA content (*P* < 0.05), likely due to their stimulation of LAB growth. Notably, both GA and TA3 treatments increased higher LA content compared to CK (*P* < 0.05). This phenomenon may be attributed to the inhibitory effects of GA and high TA concentrations on pathways that metabolize LA.

The NH_3_-N content serves as an indicator of protein degradation; a lower NH_3_-N content signifies decreased amino acid deamination and decarboxylation within the silage, which is indicative of excellent fermentation conditions [45]. Our research has revealed that TA and GA can reduce protein degradation losses as evidenced by lower NH₃-N content in the silage.

During fermentation, Lactobacilli metabolize sugars into pyruvate through the glycolytic pathway (e.g., the EMP pathway), which is subsequently reduced to lactic acid by lactate dehydrogenase [66]. Moreover, LAB synthesize not only LA but also antimicrobial compounds, such as bacteriocins and hydrogen peroxide, that directly suppress or kill detrimental microorganisms [67] Silage significantly increases the number of lactic acid bacteria compared with fresh samples. With prolonged ensiling, LAB counts significantly declined (*P* < 0.05, Tables 1 and 3) in the TA group and PEG1 treatment, whereas GA2 and PEG2 treatments exhibited a notable increase (*P* < 0.05, Tables 2 and 3). Both TA and GA exhibited inhibitory effects on LAB growth during the initial 30 days of ensiling; however, this suppression diminished in later fermentation stages. By 60 days, a reversal emerged, with TA2, TA3, and GA2 groups showing significantly higher LAB populations than the CK (*P* < 0.05). This recovery may be due to reduced bioactivity of tannic acid under weakly acidic conditions, which promotes its polymerization and diminishes its antimicrobial potency [68]. In stark contrast, the PEG group consistently maintained higher LAB population (*P* < 0.05) than CK throughout the ensiling period.

The presence of mold, yeast, and aerobic bacteria negatively impacts silage preservation. Throughout the ensiling process, mold levels remained below 2.0 log CFU/g FM in all groups. Notably, the TA and GA groups exhibited significantly reduced aerobic bacterial fermentation compared to other treatments. The DM loss in silage often results from yeast activity, as yeasts metabolize soluble carbohydrates and generate ethanol as a fermentation byproduct [69]. The results demonstrate that all three treatments effectively suppressed yeast activity (*P* < 0.05, Tables 1– 3), thereby reducing dry matter loss. Notably, the GA and TA treatments achieved this through their intrinsic microbiostatic properties, whereas PEG acted indirectly by promoting LAB fermentation and subsequent lactic acid production to inhibit yeast proliferation.

During ensiling, AA content in the TA1 group was significantly higher than in CK (*P* < 0.05, Table 1), while GA1 and GA2 exhibited significantly lower AA levels after 60 days (*P* < 0.05, Table 2). As ensiling progressed, both the TA and PEG groups showed a gradual increase in AA, likely due to rising pH, which may drive the metabolic conversion of LA into AA or BA [70]. By day 60, PA levels in TA3 and GA were significantly reduced compared to CK (*P* < 0.05, Tables 1 and 2). During ensiling, dominant bacterial phyla, such as Firmicutes, utilize LA as a metabolic substrate, converting it into acetic acid, propionic acid, and other compounds [71]. This experiment demonstrated that GA treatment effectively increased LA content while reducing both AA and PA contents, likely by inhibiting microbial pathways responsible for the conversion of LA to these organic acids LA degradation into BA is widely regarded as one of the most undesirable anaerobic fermentation processes in silage, causing significant nutrient losses—up to 51% in dry matter and 8% in total energy [72]. Throughout the silage process, almost no BA was detected, indicating high-quality fermentation in this *Moringa oleifera* leaves silage and effective inhibition of harmful bacteria such as *Clostridium*.

### Additive silage improves aerobic stability

**Fig. 2:**
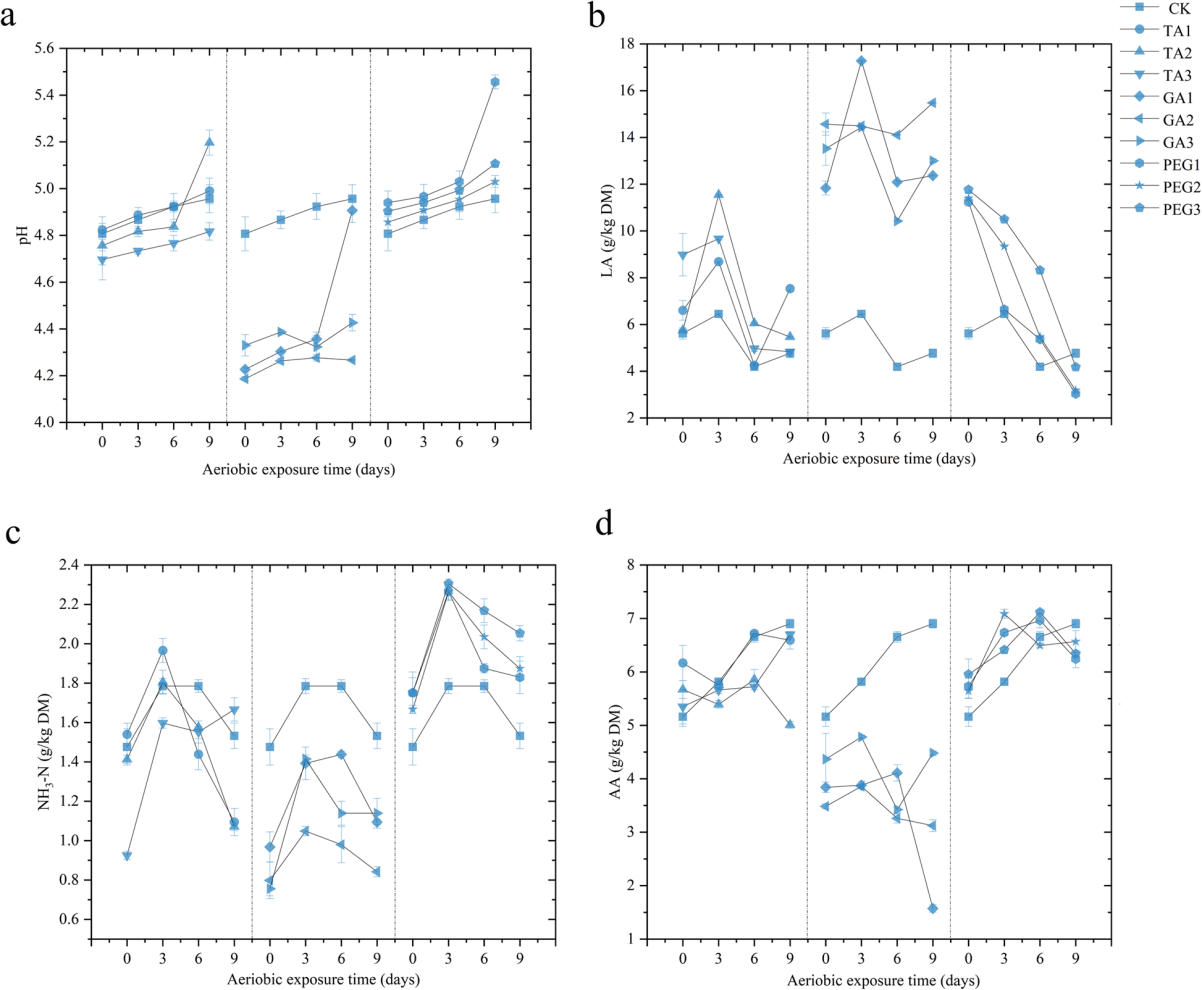
Effects of three additives on aerobic exposure quality. **a** Effects of three additives on pH. **b** Effects of three additives on LA. **c** Effects of three additives on NH_3_-N. **d** Effects of three additives on AA

When silage is exposed to air, the anaerobic environment deteriorates, leading to yeast activation. This microbial activity degrades LA and proteins, releasing CO_2_ and NH_3_-N while accelerating nutrient loss. Typically, well-fermented high-moisture silage maintains a pH of approximately 4.2, with suitable pH values generally enhancing preservation time and aerobic stability [73]. Our findings revealed that the pH of GA group during aerobic exposure remained consistently lower than that of CK (*P* < 0.05). Strikingly, the GA2 treatment maintained a stable pH (4.2) even by day 9—significantly lower than all other groups (*P* < 0.05, Fig. 2a). Meanwhile, the pH values in the CK, TA, and PEG groups exhibited a gradual upward trend over time. The rapid pH increase after aerobic exposure may reduce the resistance of *Moringa oleifera* leaves silage to deterioration. In Yuan et al.'s [74] study, aerobic spoilage was defined as a pH increase exceeding 0.5 units compared to the silage pH on day 0 of aerobic exposure. The PEG1 group surpassed this threshold, likely because the 5% PEG addition inactivated intrinsic tannins, reducing their inhibitory effect on aerobic fermentation, and its LAB content was lower than in the 10% and 20% PEG treatments. Furthermore, the aerobic stability of silage and the proliferation of secondary aerobic microorganisms during aerobic exposure are influenced by key substrates, including DM and WSC. Buffering capacity also crucially influences aerobic stability by regulating the rate of pH increase [75]. During aerobic exposure, various organic acids are produced in the feed, creating competitive pressure on yeast activity and consequently improving aerobic stability [76]. Some LA production in the feed was still detected in the CK, TA and GA groups at the beginning of the aerobic exposure. As the duration of aerobic exposure increased, fermentation by aerobic microorganisms resulted in the consumption and conversion of LA. In this experiment, LA content in the CK group stabilized with prolonged exposure, whereas the PEG group showed marked LA depletion as exposure time increased. The TA treatment had significantly higher LA content than CK after 0–3 days of aerobic exposure (*P* < 0.05, Fig. 2b). Consistent with the pH variation, the LA content in the GA group remained significantly higher than that in the CK group throughout the entire aerobic exposure period. Notably, the GA2 treatment exhibited consistently high and stable LA levels across each day of aerobic exposure. This enhancement in LA content contributed to improved aerobic stability of the *Moringa oleifera* leaves, potentially due to the inhibitory effect of GA on aerobic bacteria.

A temporary rise in NH_3_-N content was noted across all treatment groups during the 0–3 days of aerobic exposure, but the NH_3_-N content diminished as the exposure period extended. The TA1 and TA2 were effective in reducing NH_3_-N content during prolonged aerobic exposure when compared to the CK; the PEG-treatment group consistently exhibited elevated NH_3_-N concentrations. This phenomenon likely resulted from the reduced protective effect of endogenous condensed tannins on proteins. All GA-treated groups demonstrated significantly lower NH_3_-N levels compared to the control (*P* < 0.05, Fig. 2c). This is similar to the results of the study by Zhang et al. [58] using 0.2% GA addition in whole-plant maize silage, where the addition of GA may limit the deamidation of peptides or amino acids in silage, leading to the production of less NH_3_-N in the silage and enhancement of feed nutrient retention. Specifically, on days 3, 6, and 9 of aerobic exposure, the GA2 group consistently demonstrated the lowest NH_3_-N levels (*P* < 0.05), indicating the least nutritional degradation among the groups. Wang et al. [77] revealed that when 0.5% and 1%GA were used for whole-plant soybean silage, the results also showed that 1%GA could more effectively inhibit the production of NH_3_-N. which was also related to the lower pH value of 1% GA throughout the aerobic exposure period. Tao et al.'s [78] research reported that carboxypeptidase is the main exopeptidase responsible for the protein hydrolysis of silage, and its optimal pH value is 5.0. A lower pH value will inhibit its activity, thereby reducing the hydrolysis of the protein. The reason why the inhibitory effect of 2% GA on NH_3_-N was not as good as that of 1%GA might be that the high dose of GA over-bound with protein, inhibiting the metabolism of beneficial bacteria and instead leading to abnormal protein decomposition.

AA is usually the second most abundant organic acid after LA in most silages. Its content ranges from approximately 1% to 3% of the dry matter of the feed. AA gives silage its unique acidic flavor and helps to maintain its stability under aerobic conditions. It has been suggested that AA acts as an inhibitor of aerobic microorganisms and has a dose effect, with higher AA increasing the aerobic stability of silage [79]. The AA content in the CK group increased with exposure time on days 0–9 of aerobic exposure. The addition of tannin-based additives did not enhance AA production during aerobic processes. Conversely, TA2 and GA treatments exhibited reduced AA levels under aerobic conditions. Notably, the GA2 group demonstrated optimal aerobic stability alongside lower AA concentrations (Fig. 2d), attributed to the 1% GA treatment’s efficacy in suppressing the proliferation of aerobic spoilage bacteria upon oxygen exposure.

### Silage enriches the bacterial community

**Fig. 3:**
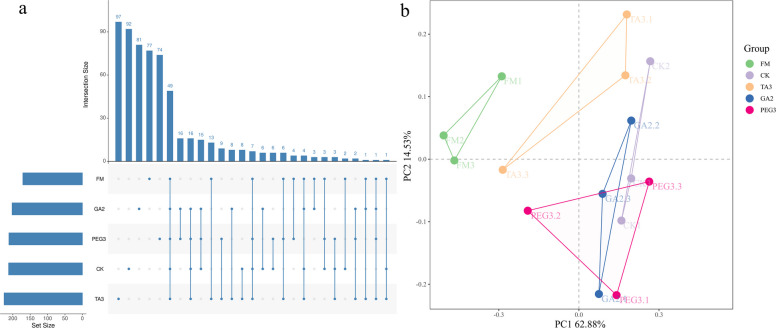
Bacterial community analysis. **a**. Upset plot of *Moringa oleifera* sinensis. **b**. Principal coordinates analysis

**Fig. 4:**
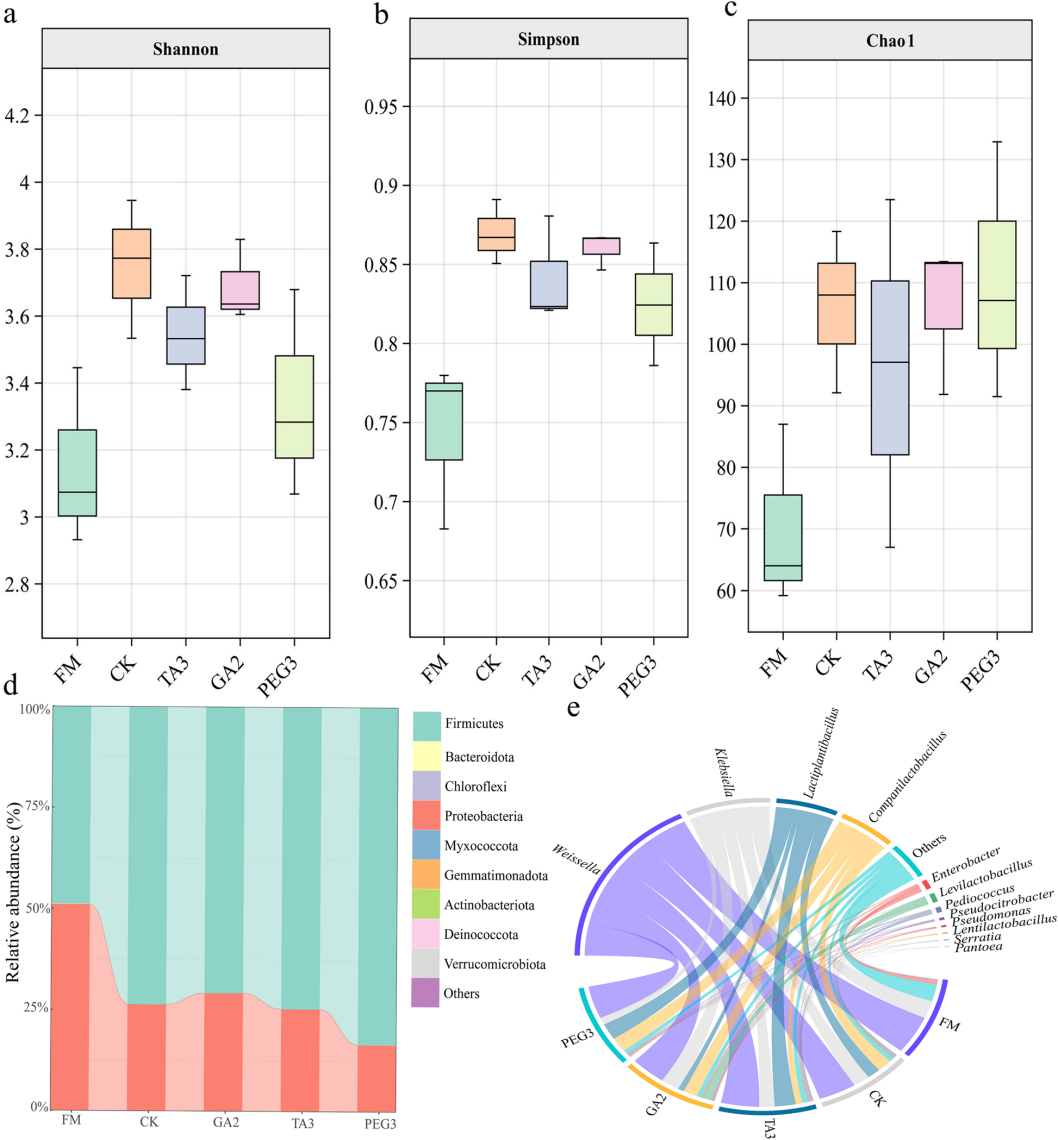
Alpha diversity and microbial community composition.** a** Shannon index. **b** Simpson index. **c** Chao1 index. **d** Bacterial community composition at phylum level. **e** Bacterial community composition at genus level

To explore how additives influence *Moringa oleifera* leaves silage, we analyzed shifts in microbial communities before and after ensiling. A total of 602 ASVs were identified within the ASV clusters. Specifically, the numbers of unique ASVs in FM, CK, TA3, GA2, and PEG3 were 45, 75, 67, 62, and 61, respectively. Additionally, the number of ASVs shared among these groups amounted to 46 (Fig. 3a). PcoA plots show a clear distinction between microbial communities before and after ensiling (Fig. 3b). Chao1, Shannon, and Simpson indices increased after silage compared to FM, indicating an increase in microbial abundance, diversity and uniformity after silage (Fig. 4a-c). The phylum Proteobacteria is widely distributed in the natural environment and in plants and animals, including many harmful fermentations bacteria, such as *Escherichia coli*. [80]. Firmicutes phylum can grow rapidly under low pH conditions, so low pH conditions in additive fortified silage may be more beneficial to the species of Firmicutes phylum [81]. After silage, we observed a marked microbial shift characterized by increased Firmicutes relative abundance corresponding with substantial depletion of Proteobacteria (Fig. 4d), among which the relative abundance of Firmicutes in the PEG3 group was higher, which was consistent with the findings of PEG addition to Neolamarckia cadamba by He et al. [62].

During the early stage of silage production, Lactococcus lactis, which mainly undergoes hetero fermentation, proliferates rapidly, initiating lactic acid fermentation and thereby reducing the pH value of the system. More acid-tolerant species, such as *Lentilactobacillus*, *Pediococcus*, and specifically *Lentilactobacillus buchneri*, commence proliferation and subsequently dominate the fermentation process. This further decreases the environmental pH, effectively inhibiting the growth and proliferation of undesirable microorganisms [82]. At the genus level, the bacterial community structure of *Moringa oleifera* leaves, both before and after ensiling, was dominated by *Weissella*, *Lactiplantibacillus*, *Klebsiella*, *Companilactobacillus*, *Levilactobacillus*, *Enterobacter*, *Pediococcus*, *Lentilactobacillus*, *Pseudomonas*, and *Pantoea* (Fig. 4e). Among them, *Lactiplantibacillus*, *Companilactobacillus* and *Weissella* are more abundant. *Lactiplantibacillus* is the most core and commonly used homopfermenting lactic acid bacteria in silage, which can rapidly ferment to produce a large amount of LA and quickly lower the pH. *Companilactobacillus*, as an atypical fermenting lactic acid bacteria, participates in LA fermentation and helps lower the pH.. *Weissella* is a beneficial bacterium in silage fermentation and produces a mixture of lactic acid and amino acids through fermentative metabolism [83]. Most *Weissella* species are specialized heterofermentative bacteria that convert WSC into lactate and acetate as the main end products [84]. Compared to the CK, TA3 and PEG3 treatments showed increased relative abundance of *Weissella*, whereas GA2 displayed a decrease-though none of these differences were statistically significant (*P* > 0.05) *Klebsiella* species are typically considered detrimental microorganisms in silage fermentation. In high-moisture silage, where *Klebsiella* is frequently detected, they have the potential to diminish the aerobic stability of the silage [85]. Analogous to the findings reported by Zou et al. [86], the relative abundance of *Klebsiella* was reduced after silage. In the present experiment, the PEG3 treatment exhibited the lowest relative abundance of *Klebsiella*. *Klebsiella* emerged as the second most dominant genus in the GA2, CK, and TA3 groups, a pattern potentially attributable to the DM and WSC content of the raw material. Additionally, TA, GA, and intrinsic tannins may suppress *Lentilactobacillus* fermentation, thereby creating favorable conditions for *Klebsiella* proliferation. This may be because the dense outer membrane structure outside the cell wall of *Klebsiella* confers greater tolerance to phenolic substances. In this experiment, the relative abundances of *Companilactobacillus and Pediococcus* ranged from 10.15% to 19.46% of the total bacterial community. Among them, *Companilactobacillus* levels in TA3 were significantly reduced compared to other groups, whereas *Pediococcus* was markedly enriched in GA2. It has been established that *Companilactobacillus* and *Pediococcus* possess the capability to inhibit the activity of high-risk antibiotic resistance genes harbored by *Enterobacter* and *Klebsiella* [87]. *Lactiplantibacillus plantarum* is a Gram-positive bacterium, and its fermentation in silage is generally homo-lactic fermentation, which produces lactic acid in the early stage of ensiling and rapidly reduces pH. In this study, compared to CK, the relative abundance of *Lactiplantibacillus* augmented in the TA3 and PEG3 treatments without achieving significance (*P* > 0.05). The trends in the relative abundance of *Lactiplantibacillus* and *Levilactobacillus* in the GA2 treatment exhibited an inverse pattern compared to those observed in other groups. This discrepancy may arise from the inhibitory effect of GA on *Lactiplantibacillus* fermentation, thereby preserving more WSC substrates for the heterofermentative fermentation by *Levilactobacillus*. It is consistent with this experiment that Wang et al. [88] also detected higher abundance of *Pseudomonas* in *Moringa oleifera* leaves silage, which further confirms that the composition of the bacterial community after silage is related to the test material itself. Previous studies have shown that condensed tannins can inhibit the activity of *Pediococcus* with little effect on the activity of *Enterobacter* [39]. This is consistent with the results of the present study.

### Correlation analysis between different indicators and bacteria

**Fig. 5:**
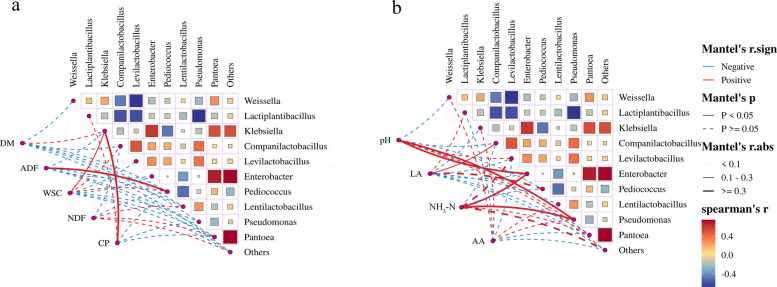
Correlation analysis between different indexes and bacterial communities, **a** Correlation analysis between nutrient index and bacterial community. **b** Correlation analysis between fermentation index and bacterial community. The triangles in the upper-right corner represent the correlation between different indicators, and the different colors indicate the spearman correlation coefficients. The lines in the lower-left corner represent the correlation of bacterial and fungal community composition with each indicator obtained through partial Mantel tests. The thickness of the lines indicates Mantel’s r statistic for the corresponding distance correlation, and dotted line and solid line represents statistical significance on the basis of different permutations

Microbial correlation analysis with physicochemical indicators revealed that *Enterobacter* and *Pseudomonas* exhibit strong positive correlations with pH and NH_3_-N concentrations (*P* < 0.05, Fig. 5b). Their proliferation competitively depletes WSC, a key substrate for LAB, thereby impeding lactic acid synthesis and delaying pH reduction. Additionally, the poor fermentation caused by them directly leads to the accumulation of NH_3_-N [89]. The diversity of the fermentation community forms a complex network of microbial interactions, in which the interactions among microorganism shape and drive the community [90]. A statistically significant positive correlation was observed between *Enterobacter* and both *Klebsiella* and *Pantoea* (*P* < 0.05). This finding led us to hypothesize that the slow decrease in pH induced by *Enterobacter* fermentation during the silage process would result in a lesser inhibition of acid-intolerant bacteria, such as *Klebsiella* and *Pantoea*. Ogunade et al. [91] found that *Pantoea* can reduce the concentration of NH_3_-N in silage, which differs from our results, and the role of *Pantoea* in the silage process needs to be investigated further.

*Lentilactobacillus* isolated from fermented vegetables such as mustard or onion leaves, as well as from fruits, sourdoughs, or related cereal ferments [92]. Its abundance was significantly and positively correlated with LA concentration (*P* < 0.05), which was attributed to the fact that *Companilactobacillus* is an important genus of lactic acid-producing bacteria in *Moringa oleifera* leaves silage fermentation. *Companilactobacillus* played an important role in pH regulation and lactic production during this silage trial. Some other studies found that the abundance of *Lentilactobacillus* was positively correlated with the concentration of acetic acid [93], *Lentilactobacillus* was positively but not significantly (*P* > 0.05) correlated with acetic acid concentration in this study. *Klebsiella* competes with LAB for substrate and producing ethanol, acetate and gas [94]. However, research has also demonstrated that *Klebsiella* species are capable of producing cellulase, thereby facilitating the degradation and conversion of lignocellulose into WSC [95]. Furthermore, Tondo's [96] research indicates that certain acid-producing *Klebsiella* bacteria possess extremely high proteolytic activity and can efficiently break down proteins. This may explain the significant correlation between *Klebsiella* and WSC and CP in this experiment (*P* < 0.05). We observed that *Klebsiella* was highly negatively correlated with *Pediococcus*, while *Pediococcus* was significantly positively correlated with the content of ADF (*P* < 0.05). This is because the lactic acid or bactercin produced by the fermentation of *Pediococcus* would have an inhibitory effect on fibro-degrading bacteria such as *Klebsiella* (*P* < 0.05).

### *TA and GA reduce methane emissions *in vitro* and do not affect dry matter digestibility*

**Fig. 6:**
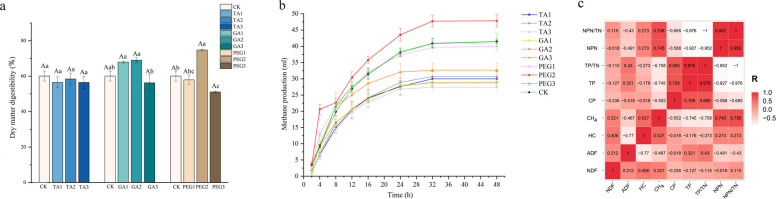
Dry matter digestibility and rumen methane production in vitro. **a** Dry matter digestibility. **b** Rumen methane production in vitro. **c** Correlation analysis of protein components, fiber components and methane production

After 60 days of ensiling, both TA and GA addition significantly reduced methane production compared to CK (*P* < 0.05, Fig. 6b). Kinetic analysis showed that TA group and GA group had lower maximum cumulative gas emissions (Table S2), and TA3 treatment (27.3 ml) and GA1 treatment (28.5 ml) were the most obvious. RFV and RFQ increased in the GA1, GA2, PEG1, and PEG2 treatment groups compared to the CK group (Table S3). Among the TA treatments, the TA3 group exhibited the lowest methane production, likely due to the stronger inhibitory effect of high-concentration TA on methanogens. However, a similar trend was not observed in the GA treatments. In the GA treatment, the GA2 group showed a higher methane yield, this is likely due to the higher HC and lower ADF content in the raw materials of the GA2 treatment group. Correlation analysis shows that HC has a positive correlation with CH_4_ (R = 0.527), while ADF has a negative correlation with CH_4_. (R = −0.467).(Fig. 6c). HC is easily degraded by rumen microorganisms, and the hydrogen produced during its degradation becomes the main energy source of methanogens, thus promoting methane production. At the same time, the higher protein content in the GA2 treatment group indicates that its acetic acid fermentation is good, which makes the GA2 treatment group have better fermentation quality and higher CH_4_ production, showing a typical dose-dependent two-way effect. Of all the treatments, the PEG2 treatment had the highest methane yield, and the methane yield of the PEG1 and PEG3 treatments was close to that of the CK group. The hydrogen gas produced by metabolism during feed fermentation is an important substrate for methanogens to reduce carbon dioxide to methane. The hydrogen gas produced by metabolism in the rumen includes gaseous hydrogen gas and dehydrogenated hydrogen gas, among which dehydrogenated hydrogen gas is easily utilized by methanogenic bacteria. Existing studies have revealed that methanogenic bacteria can influence methane production through the transfer of dehydrogenated hydrogen gas with related protozoa [97]. Dai's research indicates that the methane production decreased after the addition of tannin, but the number of protozoa did not decrease [98]. This suggests that tannin-like compounds may directly act on methanogenic bacteria. We speculate that the decrease in methane production caused by GA and TA in this experiment may be attributed to their effects on methanogenic bacteria, as tannic acid can bind to the surface film of methanogenic bacteria and inhibit their growth. At the same time, it affects methane production as a hydrogen sink. Studies further demonstrate that tannins form protein-binding complexes on methanogenic bacterial surfaces, effectively suppressing their metabolic activity [99]. The analysis revealed no significant difference in dry matter digestibility among the groups subjected to various TA treatments (*P* > 0.05). The addition of GA did not affect the dry matter digestibility of *Moringa oleifera* leaves after silage (*P* > 0.05) as compared to that of CK. In our study, IVDMD is significantly higher (*P* < 0.05, Fig. 6a, b) in the 10% PEG treatment compared to other groups, demonstrating that PEG effectively neutralized intrinsic tannins. In contrast, 5% PEG addition showed IVDMD levels similar to the CK, suggesting this concentration was insufficient to fully counteract tannin inhibition. While the 20% PEG treatment completely bound tannins, it significantly reduced IVDMD (*P* < 0.05), likely due to excessive PEG interfering with ruminal microbial activity or substrate availability. Overall, in vitro experiments have shown that tannin additives used in Moringa oleander leaf silage can indeed effectively inhibit rumen methane emissions in ruminants. However, the results of this experiment still lack support from field experiments. More thorough animal experiments should be conducted in subsequent studies to verify the results.

### Functional prediction of bacterial regulation of metabolic pathways

**Fig. 7:**
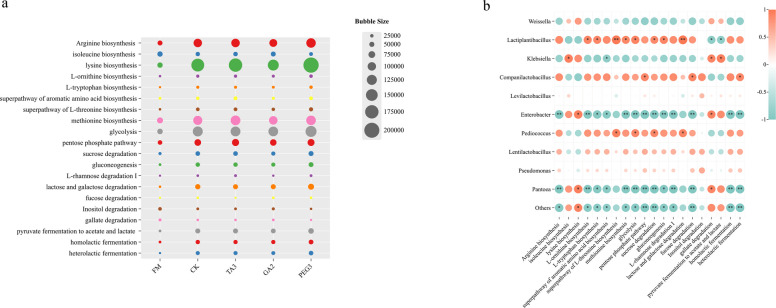
Bacterial function prediction** a** Function prediction **b** The correlation between pathway and bacteria

KEGG is a bioinformatics resource used to gain an understanding of the functions and uses of cells and organisms at the genomic level. Therefore, KEGG analysis based on Tax4Fun was conducted to predict the functional characteristics and metabolic pathways of the microbial community, with the aim of accurately assessing its role in influencing the quality of silage [100]. The prediction results showed that Silage leads to an increase in amino acid metabolic pathways, including arginine biosynthesis, methionine biosynthesis, lysine biosynthesis, L-ornithine biosynthesis, L-tryptophan biosynthesis, the super pathway of aromatic amino acid biosynthesis, and the super pathway of L-threonine biosynthesis (Fig. 7a), but compared with CK, GA2 treatment significantly inhibited the increase in amino acid metabolic pathways. This effect is not only related to the preservation of proteins, but also to its acidic environment and the unique antibacterial properties of GA. GA2 treatment simultaneously led to the down-regulation of pathways such as glycolysis, the pentose phosphate pathway, sucrose degradation, lactose and galactose degradation, gluconeogenesis, and L-rhamnose degradation. These pathways were positively correlated with *Lactiplantibacillus* and *Pediococcus*, but negatively correlated with *Enterobacter*, *Klebsiella*, and *Pantoea* (Fig. 7b). The PEG3 treatment upregulated glycolysis, pentose phosphate pathway, gluconeogenesis, and degradation of galactose, lactose, L-rhamnose, and fucose. This improvement may stem from the fact that PEG can promote the fermentation of thick-walled bacteria such as *Pediococcus*, *Lactiplantibacillus*, and *Companilactobacillus*, while inhibiting the growth of *Klebsiella* and *Enterobacter.*

## Conclusion

The addition of TA and GA enhanced NDF and HC degradation in 30 d *Moringa oleifera* leaves silage, improving TP preservation, reducing NH_3_-N production, and slowing fermentation. TA exhibited superior TP preservation compared to GA, with TA3 and GA2 groups outperforming other treatments. PEG supplementation stimulated LAB proliferation and LA production while suppressing harmful bacteria, with 5% PEG showing significantly better TP preservation than CK. Aerobic stability analysis revealed that TA improved stability during 0–3 days, while GA (particularly GA2) was most effective over 0–9 days. PEG addition compromised silage aerobic stability. Notably, both TA and GA significantly reduced in vitro CH_4_ emissions (*P* < 0.05). In our experiments, the addition of 1% GA promoted the fermentation dominated by *Levilactobacillus*, effectively reduced protein degradation, achieved better fermentation quality than other treatments, maximized aerobic stability of the feeds, and has a higher DM digestibility than the TA treatment, the TA3 treatment demonstrated the best methane reduction capacity However, the application of this conclusion in livestock production still lacks verification through field trials, and in future experiments, particular attention should be paid to the long-term effects of TA and GA treatments on the health of livestock and poultry.

## Supplementary Information

Supplementary Material 1.

Supplementary Material 2.

Supplementary Material 3.

## Data Availability

The raw sequencing data from this experiment have been uploaded to the National Center for Biotechnology Information’s database with accession numbers PRJNA1372840.

## Abbreviations

GHG: Greenhouse gas
NPN: Non-protein nitrogen
TA: Tannic acid
GA: Gallic acid
PEG: Polyethylene glycol
CT: Condensed tannins
DM: Dry matter
WSC: Water-soluble carbohydrates
ADF: Acid detergent fiber
NDF: Neutral detergent fiber
HC: Hemicellulose
TN: Total nitrogen
CP: Crude protein
TP: True protein
NH_3_-N: Ammonia nitrogen
LA: Lactic acid
LAB: Lactic acid bacteria
AB: Aerobic bacteria
AA: Acetic acid
PA: Propionic acid
BA: Butyric acid
