# Supplementary material for: Tannin-mediated improvement of Moringa oleifera silage: nutritional quality, aerobic stability, and methane mitigation
Source: BMC Plant Biol. 2026 Mar 12;26:720. doi: 10.1186/s12870-026-08507-9 (PMC13097876; doi:10.1186/s12870-026-08507-9)
Supplement: Supplementary file 1 — Supplementary Material 1. [file 12870_2026_8507_MOESM1_ESM.docx]

| Item | Content |
| --- | --- |
| Dry matter (g/kg FM) | 256.94±2.72 |
| Crude protein (g/kg DM) | 158.92±1.45 |
| pH | 5.33±0.00 |
| Water soluble carbohydrates (g/kg DM) | 73.99±0.87 |
| Crude ash (g/kg DM) | 23.20±0.07 |
| Ether extract (g/kg DM) | 97.90±0.08 |

Table1 Moringa raw material composition analysis (Dry matter basis).
