# Supplementary material for: Tannin-mediated improvement of Moringa oleifera silage: nutritional quality, aerobic stability, and methane mitigation
Source: BMC Plant Biol. 2026 Mar 12;26:720. doi: 10.1186/s12870-026-08507-9 (PMC13097876; doi:10.1186/s12870-026-08507-9)
Supplement: Supplementary file 2 — Supplementary Material 2. [file 12870_2026_8507_MOESM2_ESM.docx]

|  | CK | TA1 | TA2 | TA3 | GA1 | GA2 | GA3 | PEG1 | PEG2 | PEG3 |
| --- | --- | --- | --- | --- | --- | --- | --- | --- | --- | --- |
| V_(∞)_ | 41.22 ± 0.96 | 29.74± 0.62 | 30.41± 0.78 | 27.34 ± 0.45 | 28.50± 0.60 | 32.22 ± 0.59 | 28.60± 0.55 | 39.17 ± 0.95 | 47.63± 1.22 | 40.97 ± 1.23 |
| X_c_ | 6.75 ± 0.61 | 6.39± 0.55 | 6.00 ± 0.70 | 6.05± 0.45 | 5.85 ± 0.59 | 4.81 ± 0.62 | 5.59 ± 0.55 | 5.73 ± 0.67 | 6.88 ± 0.67 | 6.27± 0.80 |
| K | 0.16± 0.02 | 0.18 ± 0.02 | 0.18± 0.03 | 0.20 ± 0.02 | 0.20 ± 0.03 | 0.26 ± 0.04 | 0.21 ± 0.03 | 0.19 ± 0.03 | 0.16 ± 0.02 | 0.17± 0.03 |

Table 2 In vitro gas emission kinetics of MOL silages.

V(t) is the cumulative gas emission (ml); V_（∞）_is the maximum cumulative gas emission (ml); K is the maximum gas emission rate (ml/h); X_c_ is the lag time (h);t is the time (h).
