# Supplementary material for: Tannin-mediated improvement of Moringa oleifera silage: nutritional quality, aerobic stability, and methane mitigation
Source: BMC Plant Biol. 2026 Mar 12;26:720. doi: 10.1186/s12870-026-08507-9 (PMC13097876; doi:10.1186/s12870-026-08507-9)
Supplement: Supplementary file 3 — Supplementary Material 3. [file 12870_2026_8507_MOESM3_ESM.docx]

Table 3 Analysis of Quality Parameters of MOL after 60 Days of Silage with TA, PA and PEG.

| Item | Treatment |  | *P*-value |
| --- | --- | --- | --- |
|  |  |  |  |
| RFV | CK | 108.79±0.76 | *P*＜0.05 |
|  | TA1 | 109.24±0.22 |  |
|  | TA2 | 107.58±0.97 |  |
|  | TA3 | 106.95±0.47 |  |
|  | GA1 | 113.74±0.84 |  |
|  | GA2 | 113.73±0.80 |  |
|  | GA3 | 108.20±1.4 |  |
|  | PEG1 | 116.92±0.70 |  |
|  | PEG2 | 105.09±3.8 |  |
|  | PEG3 | 118.01±2.5 |  |
| TDN | CK | 55.10±0.64 | *P*＜0.05 |
|  | TA1 | 54.04±0.26 |  |
|  | TA2 | 53.60±0.16 |  |
|  | TA3 | 52.37±0.28 |  |
|  | GA1 | 53.27±0.34 |  |
|  | GA2 | 55.53±0.18 |  |
|  | GA3 | 53.71±0.19 |  |
|  | PEG1 | 54.68±0.91 |  |
|  | PEG2 | 52.52±0.17 |  |
|  | PEG3 | 56.00±0.39 |  |
| RFQ | CK | 103.70±0.72 | *P*＜0.05 |
|  | TA1 | 104.02±0.18 |  |
|  | TA2 | 102.39±0.90 |  |
|  | TA3 | 101.65±0.47 |  |
|  | GA1 | 108.21±0.76 |  |
|  | GA2 | 108.46±0.74 |  |
|  | GA3 | 102.98±1.35 |  |
|  | PEG1 | 111.40±0.76 |  |
|  | PEG2 | 99.90±3.65 |  |
|  | PEG3 | 112.60±2.39 |  |
